# Impact of right-sided breast cancer adjuvant radiotherapy on the liver

**DOI:** 10.2478/raon-2024-0059

**Published:** 2024-11-28

**Authors:** Gonca Hanedan Uslu, Filiz Taşçı

**Affiliations:** Department of Radiation Oncology, İstinye University, Faculty of Medicine, İstanbul, Turkey; Department of Radiology, Recep Tayyip Erdogan University Faculty of Medicine, Rize, Turkey

**Keywords:** radiotherapy, right-sided breast cancer, magnetic resonance elastography, radiation-induced liver disease, liver fibrosis

## Abstract

**Background:**

In patients with right-sided breast cancer the liver can be partially irradiated during adjuvant radiotherapy (RT). We aimed to determine breast cancer RT effects on liver using with magnetic resonance elastography (MRE) and biological results.

**Patients and methods:**

This retrospective study enrolled 34 patients diagnosed with right-sided breast cancer who underwent adjuvant RT. Liver segment assessments were conducted using MRE for all participants. Additionally, a complete blood count and liver enzyme analysis were performed for each patient. All measurements were taken both prior to the initiation and upon completion of RT.

**Results:**

A statistically significant difference was found in ALT (p = 0.015), ALP (p = 0.026), total protein (p = 0.037), and albumin (p = 0.004) levels before and after RT. The highest mean liver stiffness (kPa) value was recorded in segment 8, while the lowest was observed in segment 6. A weak but statistically significant positive correlation was found between segment 5 stiffness and liver volume (p = 0.039). Additionally, a statistically significant positive correlation was detected between ALP levels and the stiffness values in segment 4A (p = 0.020) and segment 6 (p = 0.003). Conversely, a weak negative correlation was observed between the stiffness values in segment 8 and post-RT total protein levels (p = 0.031).

**Conclusions:**

MRE can help us identify the level of fibrotic stiffness in the liver segments within the RT area without establishing clinical symptoms. MRE can support the clinician in evaluating the liver functions of right breast cancer patients who underwent RT. We assume these results will facilitate new studies with a large number of patients on MRE imaging at certain intervals in the follow-up of patients with right breast cancer who received RT before the development of radiation-induced liver disease (RILD).

## Introduction

Radiotherapy (RT) is a crucial component of breast cancer treatment due to its ability to achieve local-regional cancer control and improve survival outcomes. However, certain side effects of RT may sometimes surpass the disease-related issues themselves, becoming primary determinant of patient survival.^1^

In conventional radiotherapy techniques, it is often impossible to completely protect nearby healthy organs adjacent to the irradiated volume. The more space occupied by organs with relatively low resistance to radiotherapy − referred to as “critical organs” − within the treatment area, the more severe the side effects tend to become.^2^ Critical organs include the liver and kidneys during abdominal irradiation, the intestines during pelvic irradiation, and the lenses during brain irradiation.^3,4^

In recent years, efforts have been made to mitigate the adverse patient outcomes associated with radiotherapy, especially to minimize the late side effects in left-sided breast cancer. Techniques such as intensity-modulated radiotherapy (IMRT) and deep inspiration breath hold (DIBH) are some of them. The use of these techniques has become widespread in the treatment of left-sided breast cancer, and their use for right-sided breast cancer is also recommended.^5,6^

Due to its anatomical location, the liver may be partially irradiated during adjuvant radiotherapy for patients with right-sided breast cancer.^7^ The tolerable dose for a healthy liver is generally considered to be 30 Gy. Radiation-induced liver disease (RILD) is defined in tissue exposed to doses exceeding 30–35 Gy.^8^ Therefore, liver dose restrictions are in place and are essential during abdominal radiotherapy, yet the liver is not typically regarded as an organ at risk (OAR) in breast cancer.^9^

The extent of liver damage due to irradiation can only be detected through radiological imaging techniques such as abdominal CT, ultrasound, or MRI unless clinical symptoms are present. In recent years, magnetic resonance elastography (MRE) has been increasingly used to diagnose liver diseases early. The liver MRE technique has been well described.^10^ MRE is a noninvasive technique for staging liver fibrosis with excellent reproducibility.^11^ The European and American Liver Research Associations recommend using transient elastography performed with Fibroscan^®^ to detect liver fibrosis in patients with suspected nonalcoholic fatty liver disease (NAFLD).^7^ A reliable, reproducible, non-invasive method was an unmet need to evaluate liver fibrosis. Beginning to elucidate the pathophysiology of liver fibrosis at the molecular level has made it possible to use serum markers for diagnosis. However, there is a need for another tool that will support the relationship between serum markers and histology and/or reflect histology more. Today, the best method to meet this need is transient elastography. Fibroscan^®^ is a high-tech device that numerically measures the elasticity of soft tissues with this principle. MRE could be leveraged as a diagnostic tool for evaluating chronic liver diseases to assess hepatic fibrosis. It can detect a larger portion of the liver with very good resolution in contrast with liver biopsy assessment. Additionally, MRE could be utilized as an imaging method for identifying liver fibrosis that correlates well with liver biopsy in several chronic liver diseases and NAFLD. MRE has also been shown to be superior to other noninvasive methods in assessing liver fibrosis.^12^ The risk of developing classic RILD is 5% to 35% when the entire liver is irradiated with 30 to 35 Gy.^13^ MRE is a noninvasive technique for staging liver fibrosis with excellent reproducibility. According to data obtained from previous study, liver stiffness (LS) ≤ 3 kPa is considered normal, and LS > 3 kPa is considered compatible with fibrosis.^14^ MRE is a better method for diagnosing and staging liver fibrosis as it is not influenced by factors such as obesity, ascites, inflammation, or etiology.^15^ The accuracy and reliability of MRE in diagnosing all stages of liver fibrosis, especially late-stage fibrosis and cirrhosis, have been confirmed by meta-analyses.^16^

Currently, there is limited information available regarding the late-stage effects of RT on liver function in breast cancer patients. The primary aim of this study was to assess the effects of adjuvant radiotherapy on the liver in patients with right-sided breast cancer. The secondary objective was to examine the relationship between MRE findings and biological markers in determining the extent of liver involvement due to radiotherapy.

## Patients and methods

### Study population

This retrospective and descriptive study carried out in the radiation oncology clinic of a university hospital. To work; patients with primary right-sided breast cancer who had abdominal MRE examinations within at least three months after completion of RT were selected. Patients with liver disease, patients using drugs that could damage the liver other than standard drugs used in breast cancer treatment, and patients with liver function tests (LFT) values outside the reference range before RT were not included in the study. The files of 167 patients, whose adjuvant RT was completed within one year and who were admitted to the hospital for their final follow-up, were examined retrospectively, and the study was completed with 34 patients who met the sample acceptance criteria.

### Ethical consideration

All procedures followed were in accordance with the ethical standards of the responsible committee on human experimentation (institutional and national) and with the Helsinki Declaration of 1975, as revised in 2008. Ethics committee approval has been granted from our institution with protocol number (2023/111, date 27.04.2023).

### Radiotherapy

The RT plans of all patients with breast cancer were made with the same technique and dose in our clinic. Varian R brand 13.6 version Eclipse contouring system using simulation tomographies were taken on a ToshibaR Aqullion LB model, 80 cm wide CT simulator device. Radiotherapy for all patients was planned according to standard ESTRO guidelines. After the official publication, the breast/chest wall and lymph node clinical target volumes (CTV) were delineated according to ESTRO guidelines. The planning target volüme (PTV) was cropped 2–3 mm under the skin. The prescribed dose was 50 Gy in 25 fractions (2 Gy/fraction) to the breast/chest wall and/or lymph nodes. When a breast with boost was indicated, it was delivered sequentially at 10–16 Gy doses in 5–8 fractions.

The objective was the homogenous cover of 95% of the PTV by >95% isodoses. Fifty consecutive treatment plans to the breast or chest wall with lymph node irradiation were used to calculate dose-volume histogram (DVH) values for each OAR (medullaspinalis, heart, ipsilateral-contralateral lung, contralateral breast, and liver). These dose values were classified in increasing order and divided into four quartiles. For all new treatment plans, the lower than Q2 dose constraint is now applied to each organ-at-risk to obtain optimal and sufficient beam intensity modulation to comply with clinical constraints. These dose constraints aimed to decrease the doses of OAR in patients with complex anatomy and/or irradiation volumes. Radiotherapy dose information (PTV volume, PTV mean, PTV max, liver volume, liver V30 Gy, liver mean) was recorded.

### Data collection

Liver function tests and radiological imaging results of the patients were collected from hospital records, and radiotherapy treatment dose information and liver dose information were collected from the radiation oncology clinic data archive.

### Biological hepatic function assessment

These tests are performed periodically in the hospital according to patient monitoring protocols. To evaluate liver functions, ALT, AST, GGT, LDH, ALP, Total Protein, Albumin and T. Bilirubin results were evaluated. The data of the patients before the treatment and at least 3 months after the end of the treatment were evaluated.

### Radiologic imaging

The effect of RT on the liver was evaluated with MRE. The majority of liver MREs were performed with Discovery 750-Watt MR imaging device (GE Healthcare, Chicago, IL) as a treatment position MR imaging for radiation planning. The liver MRE technique has been well described. Four axial slices were obtained through the largest cross-section of the liver. Mean liver parenchyma stiffness was calculated by averaging across manually drawn regions of interest, including only liver parenchyma, and measured by the reading radiologist. MRE results were evaluated by the second author, a radiologist. Based on previous study, LS ≤ 3 kPa was considered normal, and LS > 3 kPa was consistent with fibrosis.^6^ Patients’ liver stiffness measurement (LSM) was obtained using the W General Review program on the AW Server system. This method measured all liver segments in kilopascals (kPa) by drawing 1 cm or more from the liver edge using the free region of interest (ROI) tool to obtain measurements. Both qualitative and quantitative measurements were made. All segment measurements were made in size images that provide the best anatomical detail of the liver, avoiding the liver edge (≥1 cm from the liver edge), extra-hepatic tissues, fissures, gallbladder fossa, and large blood vessels. Values <2.5 kPa Normal, 2.5–2.9 kPa Normal or inflammation, 2.9–3.5 kPa grade 1–2 fibrosis, 3.5–4 kPa grade 2–3 fibrosis, 4–5 kPa Grade 4–5 fibrosis was considered compatible with > 5 kPa 5 fibrosis or cirrhosis. Steatosis was measured by the CAP method, expressed in decibels per meter (dB/m), and fibrosis was determined by the TE, expressed in kilopascals (kPa). All fibrosis and steatosis measurements were conducted with the Fibroscan^®^ 530 Echosens device. Measurements were taken by placing the elastography probe on the right lobe of the liver from the intercostal space (mid-axillary line, between the 9th and 11th intercostal spaces) while the patient was lying in the dorsal decubitus position with his right arm in maximum abduction. The probe used (M or XL) was selected by the automatic recommendation software on the FibroScan^®^ machine. Elastography CAP values were classified between S0–S3 based on Petroff’s scale, and elasticity (fibrosis) values were classified between F0–F4 based on Eddowes’ scale.^17,18^ Elastography was performed during abdominal MRI examinations at the patients’ last clinical follow-up.

### Statistical analysis

Data were analyzed with IBM SPSS V23. Compliance with normal distribution was examined with the Shapiro-Wilk Test. Independent Samples t-test, A paired Two-Sample t-test and Pearson Correlation Coefficient compared data with normal distribution according to binary groups. Mann Whitney U Test, Wilcoxon Test and Spearman’s rho Correlation Coefficient was used for data that did not comply with normal distribution. Analysis results were presented as mean ± standard deviation (SD) and median (minimum–maximum). The significance level was taken as p < 0.050.

## Results

The female population consisted of included 34 patients, with a median age 52.5 years (range, 32 to 68 years). Table 1 summarizes patients clinical characteristics and table 2 dosimetric data.

**TABLE 1.** Patients characteristics (N = 34)

**Characteristics**	
**Age, years**	**Mean ± SD (min-max)**
	52.53± 9.38 (32–68)
	**N(%)**
**Smoking**	
No	10 (29.4)
Yes	24 (70.6)
**Alcohol**	
No	34 (100)
**NAC**	
Yes	15(44.1)
No	19(55.9)
**Surgery**	
BCS	21(61.8)
MRM	13(38.2)
**Stage**	
1A	10(29.4)
2A	14(41.2)
2B	3(8.8)
3A	7(20.6)
**Histopathological**	
IDC	31(91.2)
ILC	1(2.9)
IMC	1(2.9)
ITC	1(2.9)

BCS = breast-conserving surgery; IDC = invasive ductal carcinoma; ILC = invasive lobular carcinoma; IMC = invasive medullar carcinoma; ITC = invasive tubuler carcinoma; MRM = modified radical mastectomy; NAC = neoadjuvant chemotherapy; RT = radiotherapy

**TABLE 2.** Dosimetric date

**Characteristics**	**Value (range)**
**RT dose (Gy)**	60.0 (50–67)
**PTV volume (cc)**	1063.35 (372.6–2389.6)
**PTV max (cGy)**	6282.4 (5323.8–7050.9)
**Liver volüme (cc)**	1436.1 (843.8–2298.1)
**Liver V30Gy (%)**	1.7 (0–12.03)
**Liver mean (cGy)**	759.6 (133.8–1699.8)

RT = radiotherapy; PTV = planing target volume

We found a statistically significant difference in the before and after RT measurements of ALT (p = 0.015), ALP (p = 0.026), total protein (p = 0.037) and albumin (p = 0.004) (Table 3). While the ALP, total protein and albumin values of the patients increased after radiotherapy; we determined that the ALT value decreased.

**TABLE 3.** Comparison of before and after radiotherapy (RT) blood parameters

	**Before RT**		**After RT**		**Test statistics**	**p-value**

	**Mean ± SD**	**Median (min - max)**	**Mean ± SD**	**Median (min - max)**		
**ALP**	79.44 ± 34.28	71,5 (33–202)	90.5 ± 53.19	82.0 (39–357)	Z = −2.232	**0.026**
**ALT**	24.84 ± 12.95	21.0 (8–62)	20.26 ± 1.,89	17.0 (8–63)	Z = −2.423	**0.015**
**AST**	23.21 ± 7.88	21.0 (13–48)	24.32 ± 12.51	21.0 (13–74)	Z = −0.089	0.929
**GGT**	35.42 ± 34.08	23.0 (7–160)	33.56 ± 30.68	24.0 (12–170)	Z = −0.241	0.809
**LDH**	228.14 ± 64.71	224.5 (108–401)	216.85 ± 45.95	210.5 (140–310)	t = 0.968	0.340
**Total protein**	71.15 ± 4.88	71.0 (51–80)	72.94 ± 3.25	72.65 (64.8–79)	Z = −2.082	**0.037**
**Albumin**	41.91 ± 2.21	42.0 (38–45)	43.36 ± 2.87	43.05 (35.6–48.7)	t = −3.12	**0.004**
**Total bilirubin**	0.52 ± 0.26	0.48 (0.11–1.3)	0.51 ± 0.29	0.46 (0.19–1.9)	Z= −0.128	0.898

ALP = alkaline phosphatase; ALT = alanine transaminase; AST = aspartate transaminase; GGT = gamma gulutamil transpeptida; LDH = lactat dehidrogenes; Z = Wilcoxon Tes; t = Paired Two Sample t Test

**FIGURE 1.**
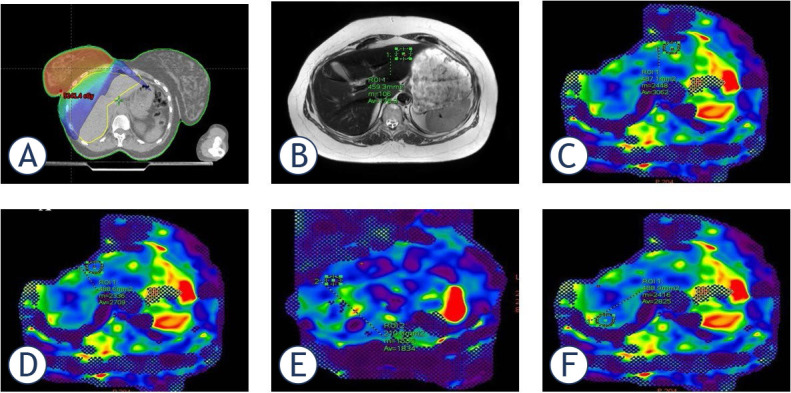
A 46-year-old female patient with a history of breast cancer and undergoing with breast-conserving surgery (BCS). **(A)** Liver distribution of radiotherapy (RT) dose (1000cGy) applied to breast; **(B)** T2 Weighted image; **(C)** Segment-2; **(D)** Segment-4A; **(E)** Segment-8; **(F)** Liver MR elastography examination from Segment-7 Elastogram measurements obtained during. When drawing the OAR, non-parenchymal structures (i.e., large vessels, bile ducts, gallbladder) that would affect the measurement were avoided. The color elastogram with a scale of 0–8 kPa shows the stiffness distribution in organs for qualitative evaluation. Orange or red regions have higher hardness values, and blue and purple regions have lower hardness values. Measured as segment-2 (3.06 Kpa), segment-4A (2.709 kPA), segment-8 (1.834 kPA), segment-7 (2.825 kPA). In the measurement made from liver segment-2, Stage 1–2 fibrosis was found. Segment-7 was normal or compatible with chronic inflammation, and the other segments were normal.

**FIGURE 2.**
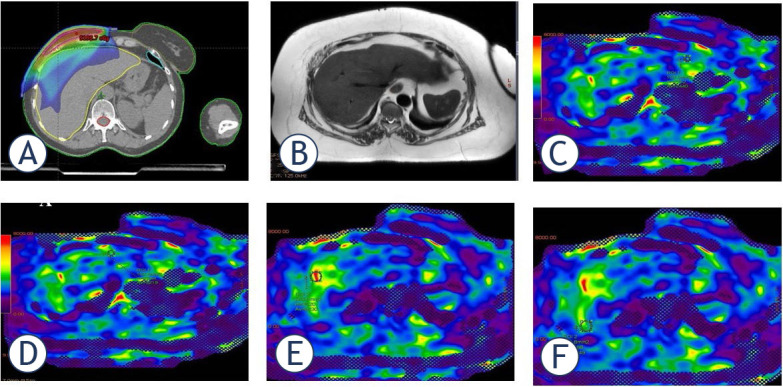
A 46-year-old female patient with a history of breast cancer who underwent modified radical mastectomy(MRM). **(A)** Liver distribution of RT dose (1000cGy) applied to chest wall location; **(B)** T2 Weighted image; **(C)** Segment-2; **(D)** Segment-4A; **(E)** Segment-8; **(F)** Liver MRE examination from segment-7 Elastogram measurements obtained during. When drawing the OAR, nonparenchymal structures (i.e., large vessels, bile ducts, gallbladder) that would affect the measurement were avoided. The color elastogram with a scale of 0–8 kPa shows the stiffness distribution in organs for qualitative evaluation. Orange or red regions have higher hardness values, and blue and purple regions have lower hardness values. Measured as segment-2 (3.44 Kpa), segment-4A (3.219 kPA), segment-8 (5.930 kPA), segment-7 (3.449 kPA). The measurement made from liver segment-2,-4A,-7 was found to be compatible with Stage 1–2 fibrosis, and the measurement from segment-8 was found to be compatible withs Stage 4 fibrosis.

**FIGURE 3.**
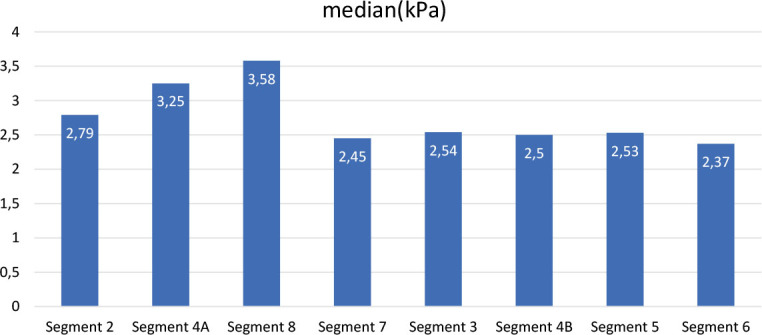
Patients kPa values in liver segments.

The mean kPa value for liver highest value segment-8 was 3.5 (range 2–5.79); the lowest value in segment-6 was 2.3 (range 1.42–3.01) (Figure 3). kPa stage rates and distributions in liver segments are shown in Table 4. The most frequent stage observed in segment-4A was 1 (38.2%) and 2 (58.8%), while in segment-8, the most common stage was 1 (26.4%) and 2 (41.1%). Also in Figures 1 and 2, examples of RT dose distributions and liver MRE images of two patients are shown.

**TABLE 4.** Distribution of kPa values in liver segments and stages (distribution of each response)

**Segment (Grade)**	**n(%)**
**Segment-2**	
1	13 (38.2)
2	14 (41.1)
3	1 (2.9)
N	6 (17.6)
N~ or Chronic Inflammation	14 (41.1)
**Segment 4a**	
1	13 (38.2)
2	20 (58.8)
3	11 (32.3)
4	2 (5.8)
N	6 (17.6)
N~ or Chronic Inflammation	3 (8.8)
**Segment 8**	
1	9 (26.4)
2	14 (41.1)
3	12 (35.2)
4	7 (20.5)
N	4 (11.7)
N~ or Chronic Inflammation	4 (11.7)
**Segment 7**	
1	2 (5.8)
2	2 (5.8)
N	19 (55.8)
N~ or Chronic Inflammation	13 (38.2)
**Segment 3**	
1	4 (11.7)
2	4 (11.7)
N	16 (47.0)
N~ or Chronic Inflammation	14 (41.1)
**Segment 4b**	
1	8 (23.5)
2	9 (26.4)
3	1 (2.9)
N	17 (50.0)
N~ or Chronic Inflammation	8 (23.5)
**Segment 5**	
1	7 (20.5)
2	7 (20.5)
N	15 (44.1)
N~ or Chronic Inflammation	12 (35.2)
**Segment 6**	
1	1 (2.9)
2	1 (2.9)
N	19 (55.8)
N~ or Chronic Inflammation	14 (41.1)

N = normal

Table 5 shows the relationship between kPa values, liver volumes, liver V30Gy and liver mean in liver segments. A weak positive correlation was found between the measurements in segment-5 and liver volume (r = 0.355; p = 0.039). Moreover, a weak but statistically significant positive correlation was observed between Segment-4A (r = 0.398; p = 0.020) and Segment-6 (r = 0.500; p = 0.003) values with ALP levels. Conversely, a weak negative correlation (r = −0.370; p = 0.031) was identified between T.PRO levels and Segment-8 values post-RT (Table 6.)

**TABLE 5.** Relationship between kPa values and liver volume in liver segments

**Liver segments (kPA values)**	**Liver volüme (cc)**	**Liver V 30Gy**	**Liver mean (Gy)**
**Segment 2**	r = 0.109; p = 0.539	r = 0.089; p = 0.616	r = 0.171; p = 0.332
**Segment 4A**	r = 0.239; p = 0.173*	r = 0.088; p = 0.619	r = −0.014; p = 0.938*
**Segment 8**	r = 0.107; p = 0.548*	r = −0.043; p = 0.809	r = −0.144; p = 0.417*
**Segment 7**	r = 0.266; p = 0.129	r = −0.139; p = 0.432	r = −0.115; p = 0.517
**Segment 3**	r = −0.068; p = 0.701*	r = −0.057; p = 0.749	r = −0.188; p = 0.286*
**Segment 4B**	r = 0.284; p = 0.103*	r = 0.224; p = 0.203	r = 0.186; p = 0.293*
**Segment 5**	**r = 0.355; p = 0.039**	r = −0.074; p = 0.679	r = −0.074; p = 0.677
**Segment 6**	r = 0.062; p = 0.729*	r = 0.073; p = 0.683	r = 0.017; p = 0.926*

R = Spearman’s rho Correlation Coefficient;

r* = Pearson Correlation Coefficient

**TABLE 6.** Relationship between after-RT segment values and liver RT dose and biochemical variables

	**Segment 2**	**Segment 4A**	**Segment 8**	**Segment 7**	**Segment 3**	**Segment 4B**	**Segment 5**	**Segment 6**
**Liver Volüm**	r=0,109; p=0.539	r=0.239; p=0.173*	r=0.107; p=0.548*	r=0.266; p=0.129	r=−0.068; p=0.701*	r=0.284; p=0.103*	**r=0.355; p=0.039**	r=0,062; p=0,729*
**Lıver V 30Gy**	r=0,089;p=0.616	r=0,088; p=0,619	r=−0,043; p=0,809	r=−0,139; p=0,432	r=−0,057; p=0,749	r=0,224; p=0,203	r=−0,074; p=0,679	r=0,073; p=0,683
**ALP**	r=0,102;p=0.565	**r=0,398; p=0,020**	r=0,175; p=0,323	r=0,298; p=0,087	r=0,149; p=0,401	r=0,227; p=0,198	r=0,002; p=0,993	**r=0,500; p=0,003**
**ALT**	r=0,145;p=0.413	r=0,165; p=0,351	r=0,246; p=0,16	r=0,259; p=0,139	r=0,172; p=0,331	r=0,163; p=0,357	r=0,107; p=0,546	r=0,294; p=0,092
**T.PRO**	r=−0,129; p=0,469	r=0,1; p=0,575*	r=−0,37; p=0,031*	r=−0,148; p=0,404	r=0,145; p=0,412*	r=0,016; p=0,929*	r=0,041; p=0,818	r=0,17; p=0,337*
**ALB**	r=0,026; p=0,882	r=0,235; p=0,18*	r=−0,113; p=0,526*	r=0,11; p=0,535	r=0,036; p=0,839*	r=0,227; p=0,196*	r=0,013; p=0,943	r=0,074; p=0,679*

ALB = albumin; ALP = alkaline phosphatase; ALT = alanine transaminase; T.PRO = total proteine; r = Spearman’s rho Korelasyon Katsayısı;

r* = Pearson Korelasyon Katsayısı

The liver volume median was 1566.6cc (range 1014–2123,5) in those with breast-conserving surgery (BCS) and 1290.6 cc (range 843,8–2298,1) in those with modified radical mastectomy (MRM). No statistically significant difference in the median V30Gy liver volume according to surgery (p > 0.05) has been achieved. The median V30Gy value was 1.9 cc in patients with BCS and 1.4 cc in patients with MRM. There was no statistically significant difference (p > 0.025) between the mean liver volume according to surgery (Table 7).

**TABLE 7.** Comparison of liver values according to surgery

	**Surgery**				**Test Statistics**	**p-value**
	**BCS**		**MRM**			

	**Mean ± SD**	**Median (min-max)**	**Mean ± SD**	**Median (min-max)**		
Liver Volume (cc)	1552.46 ± 320.52	1566.6 (1014–2123.5)	1408.3 ± 361.23	1290. (843.8–2298.1)	t = 1.214	0.233
Liver V 30Gy (%)	3.1 ± 3.03	1.9 (0–9.1)	2.8 ± 3.93	1.4 (0–12.03)	U = 111.5	0.381
Liver mean (Gy)	765.46 ± 339.81	756.7 (257.2–1564.2)	729.81 ± 451.29	762.5 (133.8–1699.8)	t = 0.262	0.795

BCS = breast conserving surgery; MRM = modified radical mastectomy; T = Independent Samples t Test; U = Mann-Whitney U-test

## Discussion

This study aimed to examine the right breast RT effect to the liver with non-invasive advanced MRE measurements and biochemical values. One of the most important aspects could be elaborated as the limited number of MRE devices in our country and the importance of data.

The liver is a sensitive organ to radiation. During ionizing radiation therapy, radiotoxicity usually accumulates in the normal tissues around the tumor, resulting in 24.7% of patients developing varying degrees of RILD.^19^ RILD is a form of subacute liver injury caused by RT. Radiation doses of 50–70 Gy are considered to be effective for controlling most solid malignant tumors; however, approximately 5–10% of patients develop classic RILD when their whole liver is exposed to the cumulative dose of 30–35 Gy.^20^ A dose of 2 Gy/day infractionated irradiation as cancer radiotherapy is sufficient to cause RILD. Patients ALP, total protein, and albumin levels increased after RT. An elevation of ALP is associated with the classical RILD.^21^ On the contrary, AST, GGT, LDH, and total bilirubin levels were not affected by radiotherapy in our study.

Liver segments 8 and 4 are the anatomical regions closest to the right breast RT region. In this study, we determined the segment with the highest kPa value. It may pose a risk for the development of RLID. During right breast radiotherapy, some specific segments of the liver had been affected more than others. Stage 2 was highest in segments-4A (58.82%) and 8 (41.18%), and the most common stage was 1–2 in segments-4A (38.2%) and segment-8 as 26.5%. Only there was a relationship between segment five measurements and liver volume values. RILD is a multi-stage, multi-step dynamic process. It links a range of responses through a complex cascade response network in which various RNAs, oxidative stress, inflammation, aging, fibrosis, and immune responses interact under the regulation of multiple signaling pathways. Alleviating tissue damage, restoring cell homeostasis, eliminating inflammation, and reducing cytotoxicity are essential for treating RILD.^22^

In this study, we determined that there was liver stiffness with MRE without deteriorating serum markers. Serum biomarkers have also been explored for liver fibrosis evaluation, but their lack of specificity poses a challenge as they may also be released during inflammation in other tissues.^23^ MRE has also been shown to be superior to other noninvasive methods in assessing liver fibrosis.^24^ MRE can also be used in the follow-up of NAFLD patients non-invasively. A recent study showed a 15% increase in MRE-LSM (liver stiffness measurement) is the strongest predictor of progression to advanced fibrosis in patients with NAFLD.^25^ Tamaki *et al*. also proposed that a combination of MRE with FIB-4 score (MEFIB index) be used for detecting patients with NAFLD and significant fibrosis for enrollment in NASH clinical trials.^26^ Beyond that, MRE-LSM is shown to be a significant predictor of the development of cirrhosis, as baseline LSM is predictive of the development of liver-related events such as decompensation or death.^27^ A recent study that evaluated the MEFIB index showed excellent negative predictive value for hepatic decompensation in patients with NAFLD-related cirrhosis. In this study, the investigators also observed that MRE-LSM is associated with hepatic decompensation, hepatocellular carcinoma, and death in patients with NAFLD-related cirrhosis.^28^

Regarding the outcomes of this research, no significant difference was observed between BCS and MRM surgery according to the liver radiotherapy doses applied to patients with right breast cancer. Although one could expect that individuals who had undergone MRM could be affected more than BCS patients as the ratio of radiation exposed volume was higher, no significant difference has been observed. However, this might be attributed to the low level of liver V30Gy in the MRM group. Also, no statistically significant difference existed in mean liver volume according to surgery; additionally, no statistically significant difference in the median 30Gy liver volume according to surgery has been achieved.

### Limitations

This study is not without its limitations. One significant limitation is the relatively small sample size, which may restrict the generalizability of the findings. Future studies with larger samples would provide more robust and reliable conclusions. Another limitation arises from the heterogeneity of the patient population, as the study included individuals at varying stages of their condition. While this may offer a broader perspective, it also introduces variability that could potentially affect the consistency of the results. Addressing these limitations in future research would strengthen the validity of the findings and provide a more comprehensive understanding of the issue. This study has some limitations. One of them is sample size. The other one is retrospective data collection and analysis, as well as a heterogeneous group.

## Conclusions

Despite the limited sample size, this study is among the few that examine the effects of breast radiotherapy on the liver using both (MRE) and biochemical markers. Regarding the outcomes of this research, MRE can help us identify the level of fibrotic stiffness in the liver segments within the RT area without establishing clinical symptoms. MRE can support the clinician in evaluating the liver functions of right breast cancer patients who underwent RT. We assume these results will facilitate new studies with a large number of patients on MRE imaging at certain intervals in the follow-up of patients with right breast cancer who received RT before the development of RILD.
